# Supplementary material for: Transdermal Flunixin Meglumine as a Pain Relief in Donkeys: A Pharmacokinetics Pilot Study
Source: Metabolites. 2023 Jun 21;13(7):776. doi: 10.3390/metabo13070776 (PMC10383245; doi:10.3390/metabo13070776)
Supplement: Supplementary file 1 [file metabolites-13-00776-s001.zip › metabolites-2410305-supplementary.pdf]

metabolites

Transdermal Flunixin Meglumine as a Pain Relief in Donkeys: A Pharmacokinetics Pilot Study

Amy K. McLean [1,2,3,*], Tara Falt [1], Essam M. Abdelfattah [4,5,*], Brittany Middlebrooks [6], Sophie Gretler [7], Sharon Spier [8], David Turoff [3], Francisco Javier Navas Gonzalez [2,9] and Heather K. Knych [7]

1 Department of Animal Science, University of California Davis, Davis, CA 95616, USA; tdfalt@ucdavsi.edu
2 World Donkey Breeds Project, Faculty of Veterinary Sciences, University of Córdoba, 14071 Córdoba, Spain; fjng87@hotmail.com
3 Equitarian Initiative, Stillwater, MN 55028, USA; dturoff@fmvs.biz
4 Department of Animal Hygiene, and Veterinary Management, Faculty of Veterinary Medicine, Benha University, Moshtohor 13736, Egypt
5 Department of Population Health & Reproduction, School of Veterinary Medicine, UC Davis, Davis, CA 95616, USA
6 Department of Clinical Sciences, Colorado State University, Fort Collins, CO 80523, USA; brittany.middlebrooks@colostate.edu
7 K.L. Maddy Equine Analytical Pharmacology Lab, School of Veterinary Medicine, UC Davis, Davis, CA 95616, USA; srgretler@ucdavis.edu (S.G.); hkknych@ucdavis.edu (H.K.K.)
8 Department of Medicine and Epidemiology, School of Veterinary Medicine, UC Davis, Davis, CA 95616, USA; sjspier@ucdavis.edu
9 Department of Genetics, Veterinary Sciences, University of Cordoba, 14071 Córdoba, Spain
* Correspondence: acmclean@ucdavis.edu (A.K.M.); eabdelfattah@ucdavis.edu (E.M.A.)

Supplementary Tables S1 and S2 report a summary for individual specific curve shape parameters, dispersion statistics, confidence intervals and determination coefficients for each administration route for Flunixin and 5OH Flunixin for each of the donkeys.

Parametric Assumptions Testing and Approach Decision

A gross violation of normality assumption occurred in Flunixin and 5OH Flunixin concentrations across all administration routes (P 0.01). Homoscedasticity was violated as well ($P<0.01$); hence, a Bayesian approach was suggested.

Bayesian Paired T-Test to Detect Differences in the Mean across Administration Routes

No significant differences (P>0.05) for almost the whole course described by Flunixin concentrations across the three administration routes, except for the late term (3rd – 4th percentile of time) of the curve, that is towards the end of the Flunixin concentration detection. While a highly significant (P<0.01) higher concentration of 0.5323 was reported when intravenous and oral Flunixin concentrations were compared, of 8.2761 higher in transdermal when compared to oral Flunixin concentrations and 7.7438 higher Flunixin transdermal concentrations than intravenous concentrations towards the 3rd percentile (75% of the time after administration) a 29.7878 higher oral Flunixin concentrations than transdermal towards the fourth percentile of the curve (100% of the time after administration).

Tables S1 presents a summary of the outputs of Related-Sample T Test to detect differences in the mean for Flunixin Concentrations Curve Shape Parameters across administration routes.

Table S1. Summary of results for Related-Sample T Test to detect differences in the mean for Flunixin Concentrations Curve Shape Parameters across administration routes.

Curve Shape Parameter	Administration Route Comparison	Mean Difference	Std. Deviation	Std. Error Mean	Bayes Factor	t	df	Sig.(2-tailed)
a	Intravenous Flunixin - Oral Flunixin	0.000	0	0	2.15	-1.059	5	0.338
a	Oral Flunixin - Transdermal Flunixin	0.000	5.6E-08	2.29E-08	1.665	1.35	5	0.235
a	Intravenous Flunixin - Transdermal Flunixin	0.000	5.6E-08	2.29E-08	1.665	1.35	5	0.235
b	Intravenous Flunixin - Oral Flunixin	0.000	2E-10	1E-10	2.046	1.118	5	0.314

b	Oral Flunixin - Transdermal Flunixin	0.000	1.143E-05	4.6664E-06	1.488	-1.47	5	0.202
b	Intravenous Flunixin - Transdermal Flunixin	0.000	1.143E-05	4.6663E-06	1.488	-1.47	5	0.202
c	Intravenous Flunixin - Oral Flunixin	0.000	1.133E-07	4.63E-08	1.826	-1.249	5	0.267
c	Oral Flunixin - Transdermal Flunixin	0.001	0.00082825	0.00033813	1.426	1.514	5	0.19
c	Intravenous Flunixin - Transdermal Flunixin	0.001	0.0008282	0.00033811	1.426	1.514	5	0.19
d	Intravenous Flunixin - Oral Flunixin	0.000	3.4954E-05	1.427E-05	1.368	1.557	5	0.18
d	Oral Flunixin - Transdermal Flunixin	-0.028	0.03623842	0.01479427	0.978	-1.894	5	0.117
d	Intravenous Flunixin - Transdermal Flunixin	-0.028	0.03621604	0.01478514	0.978	-1.894	5	0.117
e	Intravenous Flunixin - Oral Flunixin	-0.005	0.0051959	0.00212122	0.621	-2.342	5	0.066
e	Oral Flunixin - Transdermal Flunixin	0.689	0.69023638	0.28178782	0.561	2.444	5	0.058
e	Intravenous Flunixin - Transdermal Flunixin	0.684	0.68585253	0.27999812	0.562	2.442	5	0.059

f	Intravenous Flunixin - Oral Flunixin	0.532	0.3069115	0.12529609	0.111	4.249	5	0.008
f	Oral Flunixin - Transdermal Flunixin	-8.276	5.49908779	2.24499319	0.176	-3.686	5	0.014
f	Intravenous Flunixin - Transdermal Flunixin	-7.744	5.21891688	2.13061389	0.184	-3.635	5	0.015
g	Intravenous Flunixin - Oral Flunixin	-13.237	13.3151215	5.4358756	0.566	-2.435	5	0.059
g	Oral Flunixin - Transdermal Flunixin	29.788	20.3073586	8.29044445	0.19	3.593	5	0.016
g	Intravenous Flunixin - Transdermal Flunixin	16.551	17.596358	7.18368306	0.646	2.304	5	0.069

In the case of 5OH Flunixin, no differences are found up to the late end after administration (3rd percentile, 75% of post administration time) with transdermal concentrations of 5OH Flunixin being 32.2570 higher than intravenous and transdermal concentrations being 33.0556 than oral concentrations. However, towards the 4th percentile (100% proadministration time) transdermal concentrations suddenly decreases with highly significant differences ($P<0.01$) being found in 5 OH Flunixin concentrations between transdermal and either intravenous or oral administration routes, with intravenous concentrations being 34.4196 higher than transdermal and 35.041 oral concentrations being higher than transdermal concentrations, respectively.

Table S2 presents a summary of the outputs of Related-Sample T Test to detect differences in the mean for 5OH Flunixin Concentrations Curve Shape Parameters across administration routes.

metabolites

Table S2. Summary of results for Related-Sample T Test to detect differences in the mean for 5OH Flunixin Concentrations Curve Shape Parameters across administration routes.

Curve Shape Parameter	Administration Route Comparison	Mean Difference	Std. Deviation	Std. Error Mean	Bayes Factor	t	df	Sig.(2-tailed)
a	5OH Flunixin Intravenous - 5OH Flunixin Oral	0	1E-09	4E-10	2.252	-1.001	5	0.363
a	5OH Flunixin Intravenous - 5OH Flunixin Transdermal	4.45E-07	6.411E-07	2.617E-07	1.186	1.702	5	0.150
a	5OH Flunixin Oral - 5OH Flunixin Transdermal	4.46E-07	6.407E-07	2.616E-07	1.183	1.704	5	0.149
b	5OH Flunixin Intravenous - 5OH Flunixin Oral	0.00000015	3.672E-07	1.499E-07	2.249	1.002	5	0.362

b	5OH Flunixin Intravenous - 5OH Flunixin Transdermal	-0.00817515	0.01268072	0.00517688	1.339	-1.579	5	0.175
b	5OH Flunixin Oral - 5OH Flunixin Transdermal	-0.0081753	0.01268093	0.00517697	1.339	-1.579	5	0.175
c	5OH Flunixin Intravenous - 5OH Flunixin Oral	-9.8E-08	2.688E-07	1.097E-07	2.448	-0.891	5	0.414
c	5OH Flunixin Intravenous - 5OH Flunixin Transdermal	0.18016671	0.27583796	0.11261037	1.312	1.6	5	0.171
c	5OH Flunixin Oral - 5OH Flunixin Transdermal	0.1801668	0.27583811	0.11261044	1.312	1.6	5	0.171
d	5OH Flunixin Intravenous - 5OH Flunixin Oral	0.00149488	0.00364308	0.00148728	2.244	1.005	5	0.361
d	5OH Flunixin Intravenous - 5OH Flunixin Transdermal	-1.95035543	2.91316253	1.18929362	1.261	-1.64	5	0.162

d	5OH Flunixin Oral - 5OH Flunixin Transdermal	-1.9518503	2.91444737	1.18981816	1.261	-1.64	5	0.162
e	5OH Flunixin Intravenous - 5OH Flunixin Oral	-0.05433333	0.12344823	0.05039753	2.115	-1.078	5	0.330
e	5OH Flunixin Intravenous - 5OH Flunixin Transdermal	11.131	14.8324259	6.0553125	1.035	1.838	5	0.125
e	5OH Flunixin Oral - 5OH Flunixin Transdermal	11.1853333	14.864168	6.06827116	1.03	1.843	5	0.125
f	5OH Flunixin Intravenous - 5OH Flunixin Oral	0.79866667	1.5843508	0.64680851	1.849	1.235	5	0.272
f	5OH Flunixin Intravenous - 5OH Flunixin Transdermal	-32.257	30.399594	12.4105823	0.481	-2.599	5	0.048
f	5OH Flunixin Oral - 5OH Flunixin Transdermal	-33.0556667	30.6524789	12.5138221	0.461	-2.642	5	0.046

g	5OH Flunixin Intravenous - 5OH Flunixin Oral	-0.62183333	8.78499264	3.58645823	3.43	-0.173	5	0.869
g	5OH Flunixin Intravenous - 5OH Flunixin Transdermal	34.4196667	18.2647033	7.45653391	0.084	4.616	5	0.006
g	5OH Flunixin Oral - 5OH Flunixin Transdermal	35.0415	23.0794514	9.42214658	0.171	3.719	5	0.014

metabolites

Table S3. Summary for Bayesian ANOVA outputs to evidence the existence of differences in the mean of PKa related parameters across administration routes.

Parameter	Lambda_z (1/h)	Lambda_z_lower (h)	Lambda_z_upper (h)	HL_Lambda_z (h)	Tmax (h)	Cmax (ng/mL)	AUClast (h*ng/mL)	AUCINF (h*ng/mL)	AUC_%Extrap ({%})
Between groups sum of squares	0.036	116.583	9984	580.687	109.843	31755	2201	2127	33.97
BG df	2	2	2	2	2	2	2	2	2
BG Mean Square	0.018	58.292	4992	290.344	54.922	1587	11008	10635	16.985
F value	41.867	0.397	60	117.178	181.393	188.788	4.72	4.539	38.618
Sig.	0.00	0.68	0.00	0.00	0.00	0.00	0.03	0.03	0.00
Bayes Factor	38639.232	0.071	351619.413	26360769.7	48706	63767	1.76	1.579	23876.27
Within G Sum of Squares	0.006	2201.417	1248	37.167	4.542	12615684.1	349867846	351425230	6.597
WG df	15	15	15	15	15	15	15	15	15
WG Mean Square	0.00	146.76	83.2	2.478	0.303	841045.606	23324523	23428348.7	0.44
Mean Intravenous	0.14	19.25	40	5.118	0.08	9839.433	10632.167	10641.667	0.085
95% Credible Interval Intravenous	0.118-0.154	8.708-29.792	32.063-47.937	0.477-3.749	-0.399-0.559	9041.422-10637.445	6429.685-14834.648	6429.842-14853.491	-0.492-0.662
Mean Oral	0.14	13.58	56	5.423	1.333	1976.8	12583.5	12589.167	0.077

95% Credible Interval Oral	0.117-0.153	3.042-24.125	48.063-63.937	0.477-4.054	0.855-1.812	1178.788-2774.812	8381.018-16785.982	8377.342-16800.991	-0.5-0.654
Mean Transdermal	0.041	18.667	96	17.317	5.833	161.467	4383.667	4521	2.995
